# Risk Factors Affecting Cognitive Impairment of the Elderly Aged 65 and Over: A Cross-Sectional Study

**DOI:** 10.3389/fnagi.2022.903794

**Published:** 2022-06-16

**Authors:** Fengyue Han, Changjiang Luo, Duojiao Lv, Long Tian, Chuanqiang Qu

**Affiliations:** ^1^Department of Neurology, Shandong Provincial Hospital, Shandong University, Jinan, China; ^2^Department of Neurology, Jinan Shizhong District People’s Hospital, Jinan, China; ^3^Department of Neurology, Shandong Provincial Hospital Affiliated to Shandong First Medical University, Jinan, China

**Keywords:** cognitive dysfunction, aged, risk factors, logistic regression, cross-sectional

## Abstract

**Objectives:**

Elderly population with cognitive impairment has been accelerating in China. This study aimed to explore the relationship between each risk factor and each cognitive domain to provide evidence for risk prevention of controlling impaired cognitive function in elderly.

**Methods:**

This cross-sectional study analyzed the cognitive status of the elderly aged 65 and above in three communities in Shizhong District of Jinan City. Cognitive status was assessed by MMSE. The influencing factors of cognitive impairment were analyzed by chi square test, correlation analysis and regression analysis.

**Results:**

Among 1,171 participants, 643 were defined as cognitive impairment with an incidence of 54.9%. And we found that there were significant differences in the incidence of cognitive impairment among residents with different gender, age, education level, hypertension and LDL-C (*P* < 0.05). However, BMI, marital status, smoking, physical exercise, T2DM, TC, TG and HDL-C had no significant differences in the incidence of cognitive impairment. In addition, education level (*b* = 1.194, *P* <0.001), age (*b* = −0.040, *P* = 0.001), LDL-C (*b* = 0.169, *P* = 0.018) had statistical significance on the total score of MMSE according to binary logistic regression analysis.

**Conclusion:**

Gender, age, education level, hypertension and LDL-C had significant differences in the incidence of cognitive impairment. And these risk factors could provide a basis for the early screening and intervention of cognitive impairment in the elderly.

## Introduction

Due to the continuous development of medical technology and advancements in nursing approaches, the number of elderly people aged 65 and more continues to rise (Eshkoor et al., 2015). Consequently, evidences showed that the prevalence of cognitive impairment (CI) is also likely to increase (Overton et al., 2019; Pettigrew and Soldan, 2019). Cognitive impairment refers to the impairment of one or more cognitive functions, which can be divided into mild cognitive impairment (MCI) and dementia according to the severity of the dysfunction. Compared with healthy older adults, MCI patients, accompanying by subjective cognitive decline with lack of objective cognitive and impaired learning and memory, are at a higher risk of dementia, with progression rates ranging from 16% per year (Fleisher et al., 2007). On average, the previous reports revealed that every four seconds, one more individual gets dementia worldwide (WHO and Alzheimer’s Disease International, 2012).

Alzheimer’s disease (AD) is the most common type of dementia, followed by vascular dementia (Tadic et al., 2016). Vascular risk factors (VRFs), including hypertension, type 2 diabetes mellitus (T2DM), coronary artery disease (CAD), hyperlipidemia, obesity and other risk factors, can lead to stroke and then vascular dementia. The VRFs also have a significant effect in the pathogenesis of Alzheimer’s disease. Besides, increasing studies have reported that several risk factors such as gender, age, education, lifestyle and diet habits are associated with the increased risk of development of MCI (Campbell et al., 2013). However, due to different participants, regions and research methods, different conclusions may be drawn from the impact of various risk factors. In addition, different diagnostic criteria for cognitive impairment can also aggravate the differences.

Besides bringing a huge economic burden to the society and patients’ families, the emergence of cognitive impairment reduces the quality of life of patients, resulting in painful physical and psychological pressure. However, as of 2016, there are still no pharmacologic treatments for MCI that have been approved by the FDA, the European Medicines Agency or the Pharmaceuticals and Medical Devices Agency in Japan (Petersen, 2016). Thus, early intervention for cognitive dysfunction, for example changes in lifestyle such as physical exercise and cognitive and memory training, is important for the prevention of dementia. This study explored the correlation between cognitive decline and various risk factors in the elderly over 65 years old in the community to identify the population with higher MCI risk and prevent the expected social and disease burden.

## Materials and Methods

### Ethics Statement

The study was conducted according to the guidelines of the Helsinki Declaration. Ethical approval was obtained prior to the start of the study from the Ethics Committee of Jinan Shizhong District People’s Hospital. Written informed consent was obtained from all participants.

### Study Population

The samples were collected from three communities in Shizhong District of Jinan City: Weijiazhuang sub-district, Liulishan sub-district and Lingxiucheng community. According to the survey data, as of August 2016, there were about 3,000 people aged 65 and above in these three communities. The sample size was estimated according to *n* = [*Z*^2^*P* (1-*p*)]/*d*^2^. *Z* = 1.96, P referring to 8.88% of the prevalence of cognitive impairment in general Chinese communities previously reported in the literature (Kuang et al., 2020), allowable error *d* = 0.02, considering 10% invalid samples, the estimated sample size was 864 people. The study was conducted from October 2016 to June 2017. The electronic ID cards of the elderly aged 65 and above in the three communities were sorted, and the residents were selected by simple random sampling method, after which the survey was conducted. Exclusion criteria included: (1) patients with history of neuropsychiatric disorders who could not understand and/or obey the research procedures and/or follow-up; (2) patients with severe anxiety and depression (HAMD > 24, HAMA ≥ 29); (3) history of malignant tumor; (4) history of nervous system disorders causing brain dysfunction; (5) patients with severe liver or renal insufficiency (ALT > 2 times the upper limit of normal or AST > 2 times the upper limit of normal; creatinine > 1.5 times the upper limit of normal).

### Data Collection and Definition

Before the survey, the participants were informed of the content and purpose of the survey, and the survey was conducted after obtaining the written informed consent. The survey included three parts: general information questionnaire and Chinese version of Mini-mental State Examination (MMSE) (Katzman et al., 1988), physical examination, and laboratory examination.

The self-made general information questionnaire included gender, age, height, weight, marriage, education level, living habits (smoking, drinking, regular diet, and physical exercise), history of chronic diseases (including T2DM, hypertension, hyperlipidemia, and CAD), and medication of related diseases over three recent years. MMSE scale was used to evaluate the cognitive function of the elderly. The scale included 11 cognitive domains: temporal orientation, spatial orientation, immediate recall, attention, delayed recall, naming, repetition, reading, executive function, expression and drawing.

Physical examination included heart rate and blood pressure measurement, and physical examination in neurology department. Participants were asked to sit for 15 min, and then an electronic sphygmomanometer (HEM-741C, Omron, Tokyo, Japan) was used to measure their heart rate and blood pressure. Continuous measurements were taken 3 times, and the average value was used as the final result. The participants were examined by a professional neurologist.

Laboratory tests included liver function (serum glutamic oxaloacetic transaminase, serum alanine transaminase, total bilirubin), renal function (blood urea nitrogen, creatinine), blood lipid (low-density lipoprotein cholesterol, high-density lipoprotein cholesterol, total cholesterol, triglyceride), blood routine (WBC, PLT, Hb), fasting blood glucose and glycosylated hemoglobin.

The total score of MMSE was 30. Those with score less than 27 were assigned to the cognitive impairment group, and those with score more than 27 were categorized as cognitive normal group. According to the level of education, participants were divided into four groups: those without systematic and formal education were defined as illiterate; those with 1-6 years of education were defined as primary school; those with 7-12 years of education were defined as secondary school; those with more than 12 years of education were defined as university. According to body mass index (calculated by height and weight), the participants were divided into 4 groups: BMI < 18.5 was defined as emaciation, 18.5 ≤ BMI < 24 was defined as normal, 24 ≤ BMI < 28 was defined as overweight, and 28 ≤ BMI was defined as obesity. According to times of exercise in one week, the participants were divided into 3 groups: never exercise; taking exercise < 3 times/week was defined as occasional exercise; taking exercise ≥ 3 times/week was defined as regular exercise. Dyslipidemia was defined as LDL-C > 4.13 mmol/L, TG > 2.25 mmol/L, TC ≥ 6.2 mmol/L or HDL < 1.03 mmol/L (Kopin and Lowenstein, 2017). T2DM was defined as diabetes mellitus diagnosed in the past, taking hypoglycemic drugs at present, or fasting blood glucose ≥ 7.0 mmol/L this time according to the criteria of the Type 2 Diabetes Mellitus Prevention Guideline in China. Hypertension was defined as having been diagnosed with hypertension in the past, taking antihypertensive drugs or measuring blood pressure ≥ 140/80mmHg this time. Hypertension grades were defined according to 2013 ESC/ESH Guidelines for the management of arterial hypertension (Mancia et al., 2013). Controlled BP was defined as systolic blood pressure (SBP) < 140 mmHg and/or diastolic blood pressure (DBP) < 90 mmHg; Grade 1 was defined as SBP 140–159 mmHg and/or DBP 90–99 mmHg; Grade 2 was defined as SBP 160–179 mmHg and/or DBP 100–109 mmHg; Grade 3 was defined as SBP ≥ 180 mmHg and/or DBP ≥ 110 mmHg.

### Statistical Analysis

Excel office software was used to establish the database. All statistical analyses were performed with IBM SPSS Statistics for Windows version 26.0 (IBM Corp., Armonk, NY). The test level was bilateral, α = 0.05. The counting data were described by frequency or percentage. χ^2^ test was used to compare the differences between groups. The risk factors with statistical significance were analyzed by binary logistic regression. Next, the correlation between the risk factors and 11 sub-cognitive domains was analyzed by correlation analysis.

## Results

### The Relationship of Risk Factors and Cognitive Impairment in the Elderly

**FIGURE 1:**
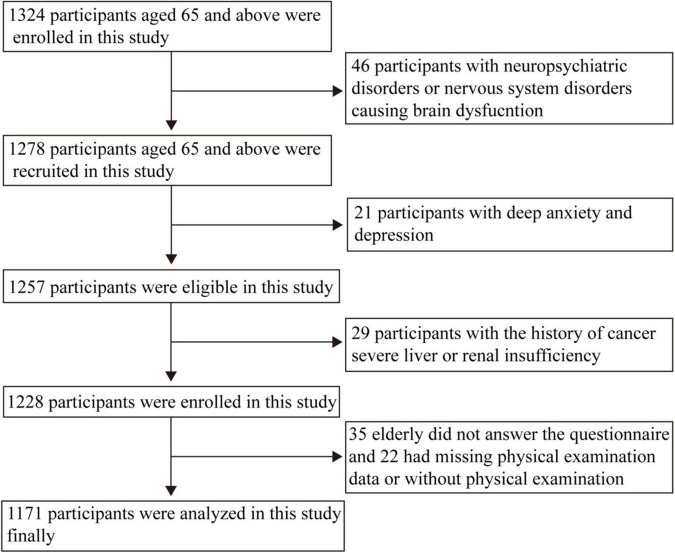
Flow diagram showing the recruitment and exclusive of the residents.

A total of 1,324 community residents were investigated in this study. After excluding invalid data, a total of 1,171 respondents entered the analysis. Details of the respondent recruitment and exclusive process in this study were showed in Figure 1. There were 528 (45.1%) with normal cognition and 643 (54.9%) with cognitive impairment.

Total 1,171 respondents included 436 men and 735 women, with a male to female ratio of 0.59:1. The respondents were aged between 65 and 95, of which 494 were aged between 65 and 69, accounting for 42.2%; 345 people aged 70-74, accounting for 29.5%; There were 332 people over the age of 75, accounting for 28.3%. Among the respondents, 171 were illiterate, accounting for 14.6%; 237 people with primary school education, accounting for 20.2%; 703 people with secondary school education, accounting for 60.0%; 60 people with university degrees, accounting for 5.1%. There were 58 people who were single, accounting for 5.0%, 896 people who were married, accounting for 76.5%; 217 people who were divorced or widowed, accounting for 18.5%. There were 434 people with smoking history, accounting for 37.1%; 520 people without smoking history, accounting for 44.4%. There were 217 people who never took exercise, accounting for 18.5%; 434 people who took exercise occasionally, accounting for 37.1%; 520 people who took exercise regularly, accounting for 44.4%; There were 12 people who were thin, accounting for 1.0%; 314 people with normal body shape, accounting for 26.8%; 544 people were overweight, accounting for 46.5%; there are 301 obese people, accounting for 25.7%. There were 956 people without type 2 diabetes, accounting for 81.6%; there were 215 people with type 2 diabetes, accounting for 18.4%. There were 691 people without hypertension, accounting for 59.0%; 317 people suffered from grade 1 hypertension, accounting for 27.1%; 137 patients with grade 2 hypertension, accounting for 11.7%; there were 26 people with grade 3 hypertension, accounting for 2.2%. There were 525 people with TC < 6.2mmol/l, accounting for 44.8%; there were 646 people with TC ≥ 6.2mmol/l, accounting for 55.2%. There were 786 people with LDL-C ≤ 4.13mmol/l, accounting for 67.1%; there were 385 people with LDL-C > 4.13mmol/l, accounting for 32.9%. There were 835 people with TG ≤ 2.25mmol/l, accounting for 71.3%; there were 336 people with TG > 2.25mmol/l, accounting for 28.7%. There were 39 people with HDL-C < 1.03mmol/l, accounting for 3.3%; there were 1,132 people with HDL-C ≥ 1.03mmol/l, accounting for 96.7%.

As shown in Table 1, there were significant differences in the incidence of cognitive impairment among residents with different gender, age, education level, hypertension and LDL-C (*P* < 0.05). There was no significant difference in the incidence of cognitive impairment among residents with different BMI, marital status, smoking history, physical exercise, T2DM, TC, TG and HDL-C (*P* > 0.05). In addition, between different age groups, the results of χ^2^-test showed that there was significant difference between 65-69 years old group and ≥ 75 years old group (*P* < 0.001), χ^2^ = 21.091; there was significant difference between 70-74 years old group and ≥ 75 years old group (*P* < 0.001), χ^2^ = 14.404; There was no significant difference between 65-69 years old group and 70-74 years old group, *P* = 0.609. Between groups with different levels of Education χ^2^ test showed that there were significant differences between the illiterate group and the primary school group, between the illiterate group and the middle school group, between the illiterate group and the university group and between the university group (*P* < 0.001), χ^2^ = 31.365,139.598,102.331; There was statistical significance between primary school group and middle school group, primary school group and university group (*P* < 0.001), χ^2^ = 54.755,36.054; There was statistical significance between middle school group and university group (*P* = 0.03), χ^2^ = 4.682. Between different blood pressure groups χ^2^ test showed that there was significant difference between normal group and grade 1 group (*P* < 0.001), χ^2^ = 14.104; there was no significant difference between the other two groups.

**TABLE 1:** Basic situation and cognitive impairment of the elderly in three communities, China, 2016.

Characteristics	Cognitive normal	Cognitive impairment	Total	χ^2^ value	*p* value
Cases, n (%)	528 (45.1)	643 (54.9)	1171		
Gender, n (%)				11.088	0.001
Male	224 (42.4)	212 (33.0)	436 (37.2)		
Female	304 (57.6)	431 (67.0)	735 (62.8)		
Age group, n (%)				23.132	<0.001
65-69years	248 (47.0)	246 (38.3)	494 (42.2)		
70-74years	167 (31.6)	178 (27.7)	345 (29.5)		
>75years	113 (21.4)	219 (34.0)	332 (28.3)		
Education group, n (%)				182.897	<0.001
Illiteracy	12 (2.3)	159 (24.7)	171 (14.6)		
Primary school	70 (13.3)	167 (26.0)	237 (20.2)		
Secondary school	403 (76.3)	300 (46.7)	703 (60.1)		
University	43 (8.1)	17 (2.6)	60 (5.1)		
Marital status				1.253	0.535
Single	25 (4.7)	33 (5.1)	58 (5.0)		
Married	412 (78.0)	484 (75.3)	896 (76.5)		
Divorced/widowed	91 (17.2)	126 (19.6)	217 (18.5)		
Smoking history				0.645	0.422
NO	313 (59.3)	396 (61.6)	709 (60.5)		
YES	215 (40.7)	247 (38.4)	462 (39.5)		
Physical exercise				5.769	0.056
Never	98 (18.6)	119 (18.5)	217 (18.5)		
Occasional	214 (40.5)	220 (34.2)	434 (37.1)		
Regular	216 (40.9)	304 (47.3)	520 (44.4)		
BMI group, n (%)				7.09	0.069
Thin group	6 (1.1)	6 (0.9)	12 (1.0)		
Normal	133 (25.2)	181 (28.1)	314 (26.8)		
Overweight	267 (50.1)	277 (43.1)	544 (46.5)		
Obese	122 (23.1)	179 (27.8)	301 (25.7)		
T2DM, *n*(%)				0.087	0.768
NO	433 (82.0)	523 (81.3)	956 (81.6)		
YES	95 (18.0)	120 (18.7)	215 (18.4)		
BP, *n* (%)				14.161	0.003
Normal	283 (53.6)	408 (63.4)	691 (59.0)		
Grade 1	170 (32.2)	147 (22.9)	317 (27.1)		
Grade 2	63 (11.9)	74 (11.5)	137 (11.7)		
Grade 3	12 (2.3)	14 (2.2)	26 (2.2)		
TC				1.916	0.166
<6.2 mmol/l	225 (42.6)	300 (46.7)	525 (44.8)		
>6.2 mmol/l	303 (57.4)	343 (53.3)	646 (55.2)		
LDL-C				5.294	0.021
<4.13 mmol/l	336 (63.3)	450 (70.0)	786 (67.1)		
>4.13 mmol/l	192 (36.7)	193 (30.0)	385 (32.9)		
TG				1.521	0.217
<2.25 mmol/l	367 (69.5)	468 (72.8)	835 (71.3)		
>2.25 mmol/l	161 (30.5)	175 (27.2)	336 (28.7)		
HDL-C				2.088	0.148
<1.03 mmol/l	22 (4.2)	17 (2.6)	39 (3.3)		
>1.03 mmol/l	506 (95.8)	626 (97.4)	1132 (96.7)		

*BMI - body mass index; T2DM - type 2 diabetes mellitus; BP-blood pressure; TC - total cholesterol; LDL-C– low-density lipoprotein cholesterol; TG– triglyceride; HDL-C– high-density lipoprotein cholesterol.*

**FIGURE 2:**
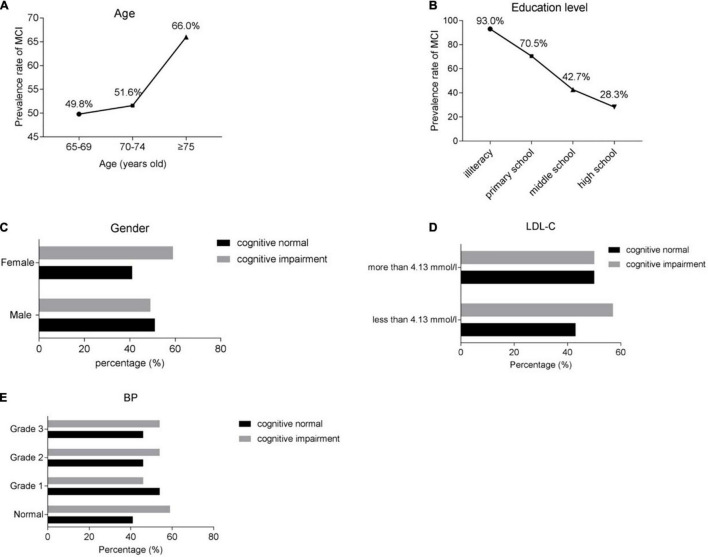
The prevalence rate of cognitive impairment in various risk factors. **(A)** The prevalence rate of cognitive impairment in different ages. **(B)** The prevalence rate of cognitive impairment in different education level. **(C)** The prevalence rate of cognitive normal or impairment in gender level. **(D)** The prevalence rate of cognitive normal or impairment in different level of LDL-C level. **(E)** The prevalence rate of cognitive normal or impairment in different level of BP level. BP, blood pressure. LDL-C, low-density lipoprotein cholesterol.

And, as revealed in Figure 2A, the respondents aged between 65 and 69, of which 246 were with cognitive impairment, accounting for 49.8%; the patients aged between 70 and 74, of which 178 were with cognitive impairment, accounting for 51.6%; the respondents aged 75 and over, of which 219 were with cognitive impairment, accounting for 66.0%, the prevalence rate of cognitive impairment was increasing with age. Among the people with illiteracy, 159 people were with cognitive impairment, accounting for 93.0%; among the people with education level of primary school, 167 people were with cognitive impairment, accounting for 70.5%; among the people with education level of middle school, 300 people were with cognitive impairment, accounting for 42.7%; and among the people with education level of high school, 17 people were with cognitive impairment, accounting for 28.3%. The result of Figure 2B demonstrated that the morbidity of cognitive impairment was decreasing with the high education level. And we showed that the people with lower LDL-C (less than 4.13 mmol/L), high grade BP and female was more likely to have cognitive impairment (Figures 2C–E).

**FIGURE 3:**
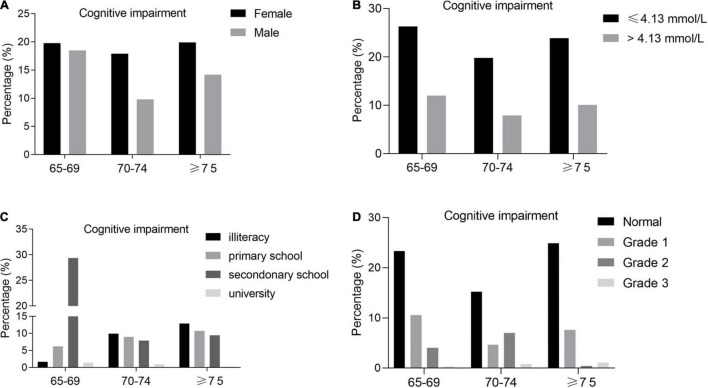
**(A)** Prevalence of cognitive impairment by gender and age group. **(B)** Prevalence of cognitive impairment by LDL-C and age group. **(C)** Prevalence of cognitive impairment by education level and age group. **(D)** Prevalence of cognitive impairment by BP and age group.

Next, we analyzed the interaction between age and gender, education level, BP, LDL-C in people with cognitive impairment. And we found that prevalence of cognitive impairment was higher among female than male and in lower LDL-C group than higher LDL-C group (Figures 3A,B). In addition, in the elderly aged 70-74 years and ≥ 75 years, the prevalence of cognitive impairment in all education level group was decreasing with aging, but the percentage of cognitive impairment in people of 65-69 years was significantly high in secondary school group perhaps due to the relatively large deviation of people number recruited to this group (Figure 3C). However, in different grade BP group, we found that the prevalence of cognitive impairment in normal group was higher than in the grade 1 group, probably the mild hypertension could improve the cognitive function through cerebral blood circulation and the like a series of positive physiological and biochemical changes (Figure 3D). And intriguingly, the effect of grade hypertension on cognitive impairment was different in the elderly aged 65-69 years, 70-74 years and ≥ 75 years maybe due to the huge differences of group people number in recruitment (Figure 3D).

The variables with statistically significant differences, including gender, age, blood pressure, education level and LDL-C, were used as independent variables for binary logistic regression analysis. The results showed that education level (B = 1.194, OR = 3.302) and LDL-C (B = 0.169, OR = 1.184) were the risk factors of cognitive impairment, while age (B = -0.040, OR = 0.961)was the protective factor. See Table 2 for details.

**TABLE 2:** Binary logistic regression of gender, age, education, blood pressure and low-density lipoprotein cholesterol on cognitive impairment, China, 2016.

	*p* value	*B* value	OR value	95% CI
Gender	0.915	–0.014	0.986	0.756-1.285
Age	0.001	–0.040	0.961	0.937-0.985
education	< 0.001	1.194	3.302	2.694-4.046
BP	0.269	0.004	1.004	0.997-1.011
LDL-C	0.018	0.169	1.184	1.030-1.361

*BP - blood pressure; LDL-C – low-density lipoprotein cholesterol.*

### The Correlation Analysis of Independent Positive Risk Factors and Sub Cognitive Domains

The variables with statistically significant differences, including gender, age, blood pressure, education level and LDL-C, were used as independent variables for correlation analysis with 11 different sub cognitive domains. The results are shown in Table 3. The results showed that there were correlations between gender and temporal orientation (*r* = 0.073), spatial orientation (*r* = 0.112), immediate memory (*r* = 0.068), attention (*r* = 0.142), reading (*r* = 0.066), expression (*r* = 0.167) and drawing (*r* = 0.094). There were correlations between age and 10 sub cognitive domains: temporal orientation (*r* = −0.082), spatial orientation (*r* = −0.131), immediate memory (*r* = −0.220), attention (*r* = −0.206), delayed memory (*r* = −0.150), naming (*r* = −0.113), repetition (*r* = −0.136), executive ability (*r* = −0.087), expression (*r* = 0.167) and drawing (*r* = 0.094). Education level was correlated with 10 sub cognitive domains: temporal orientation (*r* = 0.238), spatial orientation (*r* = 0.282), immediate memory (*r* = 0.254), attention (*r* = 0.363), delayed memory (*r* = 0.226), repetition (*r* = 0.238), reading (*r* = 0.277), executive ability (*r* = 0.172), expression (*r* = 0.500) and drawing (*r* = 0.351). There was no correlation between blood pressure, LDL-C and 11 sub cognitive domains.

**TABLE 3:** Correlation analysis of gender, age, education, blood pressure and low-density lipoprotein cholesterol on 11 sub cognitive domains in Mini-mental State Examination, China, 2016.

	Temporal orientation			Spatial orientation			Immediate recall			Attention		
	*P* value		R	*P* value		r	*P* value		r	*P* value		R
Gender	0.013		0.073	< 0.001		0.112	0.019		0.068	< 0.001		0.142
Age	0.005		–0.082	< 0.001		–0.131	< 0.001		–0.220	< 0.001		–0.206
BP	0.989		< 0.001	0.785		–0.008	0.691		–0.012	0.605		–0.015
LDL-C	0.542		0.018	0.927		–0.003	0.884		0.004	0.187		0.039
Education	< 0.001		0.238	< 0.001		0.282	< 0.001		0.254	< 0.001		0.363

	**Repetition**			**Reading**			**Executive function**			**Expression**		
	***P* value**		**R**	***P* value**		**R**	***P* value**		**r**	***P* value**		**R**

Gender	0.144		0.043	0.023		0.066	0.060		0.055	< 0.001		0.167
Age	< 0.001		–0.136	0.987		< 0.001	0.003		–0.087	< 0.001		–0.159
BP	0.276		–0.032	0.591		0.016	0.348		0.027	0.859		0.005
LDL-C	0.593		–0.016	0.187		0.039	0.329		0.029	0.158		0.041
Education	< 0.001		0.238	< 0.001		0.277	< 0.001		0.172	< 0.001		0.500

		**Delayed recall**				**Naming**				**Drawing**		
		***P* value**		**R**		***P* value**		**r**		***P* value**		**r**

Gender		0.265		–0.033		0.680		0.012		0.001		0.094
Age		< 0.001		–0.150		< 0.001		–0.113		< 0.001		–0.156
BP		0.527		–0.018		0.648		0.013		0.169		–0.040
LDL-C		0.234		–0.035		0.908		–0.003		0.955		0.002
Education		< 0.001		0.226		0.056		0.056		< 0.001		0.351

*MMSE - Mini-mental State Examination; BP-blood pressure; LDL-C – low-density lipoprotein cholesterol.*

Taking 11 different sub cognitive domains as dependent variables and age, blood pressure and LDL-C as independent variables for multiple linear regression analysis, the results are shown in Table 4. Among them, age was negatively correlated with temporal orientation, spatial orientation, immediate memory, attention, delayed memory, naming, repetition, executive ability, expression and drawing. There was no correlation between blood pressure, LDL-C and these above 11 sub cognitive domains.

**TABLE 4:** Multiple linear regression analysis of age, blood pressure and low-density lipoprotein cholesterol on 11 sub cognitive domains in Mini-mental State Examination, China, 2016.

	Temporal orientation					Spatial orientation					Immediate recall				
	B	SE	β	T	P	B	SE	β	t	P	B	SE	β	t	P
Age	–0.015	0.005	–0.082	–2.806	0.005	–0.019	0.004	–0.131	–4.525	< 0.001	–0.021	0.003	–0.220	–7.685	< 0.001
BP	< 0.001	0.002	0.002	0.065	0.948	< 0.001	0.001	–0.003	–0.094	0.925	< 0.001	0.001	–0.004	–0.131	0.896
LDL-C	0.016	0.028	0.016	0.552	0.581	–0.004	0.022	–0.005	–0.166	0.868	< 0.001	0.015	< 0.001	0.014	0.989

	**Attention**					**Delayed recall**					**Naming**				
	**B**	**SE**	**β**	**T**	**P**	**B**	**SE**	**β**	**t**	**P**	**B**	**SE**	**β**	**t**	**P**

Age	–0.060	0.008	–0.205	–7.146	< 0.001	–0.028	0.005	–0.150	–5.191	< 0.001	–0.004	0.001	–0.113	–3.894	< 0.001
BP	–0.001	0.002	–0.011	–0.380	0.704	–0.001	0.002	–0.010	–0.331	0.741	< 0.001	< 0.001	0.018	0.621	0.535
LDL-C	0.056	0.045	0.036	1.247	0.213	–0.037	0.030	–0.037	–1.262	0.207	–0.001	0.005	–0.007	–0.243	0.808

	**Repetition**					**Reading**					**Executive function**				
	**B**	**SE**	**B**	**T**	**P**	**B**	**SE**	**β**	**t**	**P**	**B**	**SE**	**β**	**t**	**P**

Age	–0.008	0.002	–0.135	–4.653	< 0.001	0.896	0.002	0.001	0.024	0.981	–0.011	0.004	–0.087	–2.994	0.003
BP	< 0.001	< 0.001	–0.025	–0.875	0.382	< 0.001	0.001	0.012	0.416	0.677	0.001	0.001	0.028	0.969	0.332
LDL-C	–0.005	0.009	–0.016	–0.541	0.589	0.017	0.013	0.037	1.276	0.202	0.016	0.019	0.024	0.832	0.406

	**Expression**								**Drawing**						
	**B**		**SE**	**B**	**t**	**P**			**B**	**SE**		**β**	**t**		**P**

Age	–0.013		0.002	–0.159	–5.490	< 0.001			–0.013	0.002		–0.154	–5.334		< 0.001
BP	< 0.001		0.001	0.008	0.260	0.795			–0.001	0.001		–0.035	–1.197		0.232
LDL-C	0.017		0.013	0.038	1.300	0.194			0.001	0.013		0.002	0.071		0.944

*MMSE - Mini-mental State Examination; BP-blood pressure; LDL-C – low-density lipoprotein cholesterol.*

## Discussion

The research results of the prevalence of cognitive impairment in the elderly over 65 years old in China have substantially changed over recent years. Kuang et al. (2020) reported that the prevalence of cognitive impairment in the elderly aged 65 years old and more in China was 8.88%, while Yang et al. (2019) indicated that the prevalence of cognitive impairment in the elderly was 15.8%. These inconsistencies may be related to different research methods, diagnostic criteria, participants, and other factors. The incidence of cognitive impairment in this cross-sectional study was 54.9%, which is much higher than that in previous studies and indicates less than optimistic cognitive status in the elderly from these three communities. Since there is no effective drug to reduce cognitive impairment, the prevention of related risk factors is very important. Medical staff must identify the related risk factors of cognitive impairment and promote targeted prevention and treatment so as to reduce the incidence of cognitive impairment in the elderly and improve their quality of life.

In this study, the elderly with cognitive impairment significantly differed in gender, age, education level, BP, and LDL-C compared with the elderly with normal cognition. Among these factors, education level and LDL-C resulted as independent risk factors for cognitive impairment, while age was a protective factor, which is completely different from the results of univariate analysis in Table 1.

Age is an important risk factor for cognitive impairment. As we age, the structure of the brain deteriorates. From a macro perspective, the incidence of brain atrophy is an inevitable consequence of aging (Liu-Ambrose et al., 2019). The atrophy of the prefrontal cortex and medial temporal lobe, which is more obvious (Salthouse, 2011), is related to executive function and naming (Harada et al., 2013). In addition, in the process of aging, the reduction of white matter volume is also very obvious. White matter has been shown to be significantly correlated with memory. In the early stage of cognitive impairment (MCI), amnesia is often the first patient complaint (Petersen, 2016). MCI is a cognitive state between normal aging and dementia (Eshkoor et al., 2015; Anderson, 2019)1 16. Eshkoor et al. believe that the diagnosis of MCI is based on the chief complaint of memory impairment (Eshkoor et al., 2015). Therefore, aging is a risk factor for extensive deterioration of multiple cognitive domains such as memory, execution, naming, attention, and reading.

Neurons’ death is the possible cause of brain atrophy (Harada et al., 2013). Neurons are non-renewable cells, so the death of neurons is very serious. Still, more theories suggest that the decline of cognitive function in the elderly is related to the decrease of the number of connections between neurons rather than the absolute number of neurons. Synapse is a common connection between neurons, and the information transmission between neurons is also based on a synapse. More studies have been suggesting that the deterioration of cognitive function is based on the decrease of synaptic density. The morphologic changes, including a decrease in the complexity of dendrite arborization, decreased dendrite length and decreased neuritic spines (the major sites for excitatory synapses), directly lead to the decrease of synaptic density (Dickstein et al., 2007).

Currently, it is generally accepted that middle-aged hypertension is related to the decline of cognitive function in old age (Hughes and Sink, 2016; Walker et al., 2017), but the effect the new onset hypertension may have on cognitive function in old age has not yet been consistently defined. It may be that the effect of hypertension on cognitive function is closely related to the duration of hypertension (Walker et al., 2017; Wei et al., 2018). It seems that this theory can be explained by the hypothesis of brain auto-regulation. When the cerebral blood flow perfusion pressure is maintained in a certain range, the cerebral blood flow can maintain a certain steady state. This function is realized by endothelial cells and vascular smooth muscle cells. If long-term persistent hypertension, the cerebral blood perfusion is relatively reduced, resulting in the reduction of the number of cerebral cortex capillaries, fibrosis, smooth muscle cell necrosis, and apoptosis (Tadic et al., 2016). Subsequently, the brain self-regulation starts to be dysfunctional, thus exacerbating the pathological process. This is a chronic, long-term and continuous process.

Hypertension is related to the decline of overall cognitive function, including memory, execution, orientation, reading, repetition, and other cognitive domains. Among these, executive function and information processing speed are the most vulnerable (Hughes and Sink, 2016; Tadic et al., 2016; Walker et al., 2017). The most common lesion associated with hypertension occurs in the frontal cortex, which is the focus of executive function and information processing. Therefore, it can be considered that hypertension affects executive function and information processing speed through the damage of the frontal cortex.

Stern proposed the hypothesis of “cognitive reserve” and defined it as the ability to optimize cognitive performance (Stern, 2002). The cognitive reserve cannot be directly calculated but can only be expressed by different indicators, among which the most commonly used is education level (Kessels et al., 2017; Mungas et al., 2018). The incidence rate of dementia is lower in individuals with higher education levels, and the average age of dementia onset is also delayed by higher education level (Xu et al., 2016). The delay of onset age means the extension of the time without cognitive impairment. To some extent, the improvement of education level improves the quality of life in the elderly. Nevertheless, it is not enough to replace cognitive reserve only by education level. Formal and systematic education years are static, while the cognitive reserve is dynamic. Skilled manual work, good reading habits, and positive social interaction can also be regarded as indicators of cognitive reserve. Moreover, the level of higher education means a better economic level and more extensive interpersonal communication, and the existence of these factors also affects the cognitive status (Kahn and Pearlin, 2006; Shin et al., 2020; Yahirun et al., 2020).

The incidence rates of cognitive impairment were higher in women compared to men (58.6 vs. 48.6%), which is consistent with previous studies (Zhang, 2006; Zhang et al., 2008). In addition to the significant differences in MMSE, there were also significant differences in time orientation, spatial orientation, immediate memory, attention, reading, expression, drawing, and other sub cognitive domains.

The elderly in this study were 65 years of age and above, and most of them were born before the founding of new China in 1949. Previous study indicated that China is a traditionally patriarchal society, where men are dominant in economic level, social status, education level, social activities, and other aspects (Miyawaki and Liu, 2019). South Korea, which has a similar history to China, also records worse cognition in older women (Cho et al., 2017). Especially before 1949, most women had no access to formal education. In addition, the longer life expectancy of women may further aggravate this difference (Hebert et al., 2001; Viña and Lloret, 2010).

LDL-C is a key factor in the formation and development of atherosclerosis and the formation of unstable plaque, which is in the central position in cardiovascular and cerebrovascular diseases (Wang et al., 2018). There are various mechanisms of LDL-C in cognitive decline. Inflammation has an important role in atherosclerosis. Oxidized low-density lipoprotein is phagocytic, forming foam cells, which is the central link of atherosclerosis, eventually leading to thrombosis and cerebral blood flow reduction, neuronal necrosis, apoptosis, and synapse reduction. It is also believed that LDL-C is related to the permeability of the blood-brain barrier and that LDL-C increases the permeability of the blood-brain barrier by reducing the expression of tight junction protein ZO-1 (Dias et al., 2015; Tanaka et al., 2018). However, this theory has not been widely accepted.

The present study has a few limitations in this study. First, this was only a cross-sectional study with a relatively small number of samples and no follow-up data. Second, some important risk factors affecting cognitive function (such as cerebrovascular disease) were not included in the regression equation. Third, our study was performed in Jinan, People’s Republic of China, where economic development, labor migration, culture, and lifestyles may influence the prevalence of CI. Finally, there are some limitations related to MMSE: MMSE has a wide range of applications, but its sensitivity is low, and some MCI may be missed. Therefore, a follow-up study needs to be performed in the future.

In conclusion, the present study revealed several risk factors to be the significant predictors of cognitive impairment. Compared with the elderly with normal cognition, the elderly with cognitive impairment has significant differences in gender, age, education level, BP, LDL-C, and so on. Among these factors, education level and LDL-C are the independent risk factors of cognitive impairment, while age is the protective factor, which is quite different from the general cognition in the past. The relationship between other risk factors such as smoking, material status, BMI, T2DM, TC, TG, HDL-C, and cognitive impairment cannot be denied. The study provided an opportunity to targeted cognitive training and intensive intervention for the elderly to delay the progression of cognitive decline.

## Data Availability Statement

The original contributions presented in this study are included in the article/supplementary material, further inquiries can be directed to the corresponding authors.

## Ethics Statement

Ethical approval was obtained prior to the start of the study from the Ethics Committee of Jinan Shizhong District People’s Hospital. Written informed consent was obtained from all participants.

## Author Contributions

CQ designed the study. FH analyzed and interpreted the data, wrote and edited the manuscript, administered the project, acquired funding, and reviewed and edited the manuscript. CL, DL, and LT contributed to the acquisition, analysis, and interpretation of the data. All authors contributed to the article and approved the submitted version.

## Conflict of Interest

The authors declare that the research was conducted in the absence of any commercial or financial relationships that could be construed as a potential conflict of interest.

## Publisher’s Note

All claims expressed in this article are solely those of the authors and do not necessarily represent those of their affiliated organizations, or those of the publisher, the editors and the reviewers. Any product that may be evaluated in this article, or claim that may be made by its manufacturer, is not guaranteed or endorsed by the publisher.

## Abbreviations

MMSE: Mini-mental State Examination
BP: blood pressure
LDL-C: low-density lipoprotein cholesterol
BMI: body mass index
T2DM: type 2 diabetes mellitus
TC: total cholesterol
TG: triglyceride
HDL-C: high-density lipoprotein cholesterol.
